# Quality of online health information about oral contraceptives from Hebrew-language websites

**DOI:** 10.1186/2045-4015-1-38

**Published:** 2012-09-24

**Authors:** Yehuda Neumark, Lior Flum, Catalina Lopez-Quintero, Ronny Shtarkshall

**Affiliations:** 1 Braun School of Public Health and Community Medicine, Hebrew University-Hadassah, PO Box 12272, Jerusalem, 91120, Israel

**Keywords:** Internet, Quality, Online health information, Content analyses, Oral contraceptives, Hebrew

## Abstract

**Background:**

The Internet is a frequently used source of health information. Adolescents in particular seem to be receptive to online health information (OHI) and often incorporate such information in their decision-making processes. Yet, OHI is often incomplete, inaccurate, or unreliable. This study assessed the quality of Hebrew online (non-user-generated) content on oral contraceptives (OC), with regard to accuracy/completeness, credibility, and usability.

**Methods:**

Twenty-nine websites in Hebrew, including those of the four Israeli HMOs, were identified and evaluated. The websites were categorized as: HMO, health portal, contraception-specific, promotional-commercial, and life style and women’s health. A set of established content parameters was selected by a family planning expert to assess accuracy/completeness. The Health on the Net Foundation Code of Conduct (HONcode) principles were used to assess the websites’ reliability. Usability was assessed by applying items selected from the Minervation Validation and the University of Michigan’s ′Website Evaluation checklist′ scale. Mean scores, standard deviations (SD), and ranges were calculated for all websites and for category-specific websites. Correlation between dimensions and Inter-rater reliability were also examined.

**Results:**

The mean score for accuracy/completeness was 50.9% for all websites (SD=30.1%, range 8–100%). Many websites failed to provide complete information, or provided inaccurate information regarding what to do when a pill is missed and when to use back–up methods. The average credibility score for all websites was 70.6% (SD=15.1, range=38=98%). The credibility parameters that were most commonly absent were funding source, authoring, date of content creation and last modification, explicit reference to evidence-based information, and references and citations. The average usability score for all websites was 94.5% (SD=6.9%, range 79–100%). A weak correlation was found between the three quality parameters assessed.

**Conclusions:**

Wide variation was noted in the quality of Hebrew-language OC websites. HMOs’ websites scored highest on credibility and usability, and contraceptive-specific websites exhibited the greatest accuracy/completeness. The findings highlight the need to establish quality guidelines for health website content, train health care providers in assisting their patients to seek high quality OHI, and strengthen e-health literacy skills among online-information seekers, including perhaps health professionals.

## Background

Despite the sometimes questionable quality of online health information (OHI) [1-3], millions of Internet users the world over seek OHI daily [4]. It is estimated that 40% to 80% of Internet users seek OHI worldwide [5-8], and considering the varying definitions of “health” the true figures may be even higher [9,10].

Oftentimes, OHI influences an individual’s knowledge, attitudes, and behaviors, and modifies the doctor-patient relationship [11-14]. For adolescents, who often thirst for health information as they cope with psychological and biological changes [15] yet are hesitant to articulate their health concerns and questions to adults [16], the Internet is a particularly attractive source of accessible and anonymous health information [17]. Adolescents are indeed among the most active users of the Internet [18], are particularly receptive to OHI, and often incorporate such information in their decision-making processes [6,19]. Worrisomely, adolescents might be particularly susceptible to the effects of erroneous health information [9] as they often lack essential e-health literacy skills [12] to critically assess the information they encounter online.

Online sources and types of OHI vary greatly and range from peer-reviewed medical journals’ websites to websites that promote risky behaviors [20], and wide gaps prevail between evidence-based medicine and the information available online [21]. The vast quantity of readily available, yet often complex, and even contradictory health information makes it difficult for the casual OHI-seeker to distinguish between true information, misinformation, and disinformation [22]. Deceptively presented OHI is not unusual, and is especially common in the presentation of search-engine results [20,23].

Websites may attempt to conceal the origin of the information (for example, by use of auto-translation software) [24] or present information of inconsistent quality and from different sources in an identical format and level of accessibility to give the false impression that all the information presented is of equal credibility [25].

Health websites also vary in their complexity, design, and aesthetics. These factors affect the user’s perceptions of website credibility (or “believability” [23]) and the effectiveness of website messages [26-28]. Research in this area suggests that the user’s judgment about credibility is formed in the first few seconds of viewing a webpage [27]. Moreover, usability (defined broadly as the extent to which the design of a website helps users find the information they are looking for [29]) is age-sensitive [30]. Adolescents and younger adults approach websites differently than older adults due to age-related differences in spatial and verbal/vocabulary abilities [22,30].

Concerns regarding the quality of OHI are not new, and various solutions aiming to control or evaluate aspects of health information quality have been developed. These range from codes of conduct to user guides, filters, and third party certification [31-34]. For example, guidelines of the Health on the Net Foundation Code of Conduct (HONcode) [35] – the oldest and most widely used code for health information, consists of eight principles, or criteria, for assessing the transparency and potential biases of OHI (e.g., authority, user data confidentiality, attribution of information to sources, and financial disclosure). Newer tools, such as the FA4CT model [36], social networks [37], and the Medication Website Assessment Tool (MWAT) [38], are continuously being added to the arsenal of information quality assessment tools. The effectiveness of many of the existing website evaluation tools remains to be proven [32,39,40].

Accuracy and completeness of OHI can be assessed by subject matter experts or by assessing the extent to which the OHI meets evidence-based practice protocols or guidelines. However, some health topics are too broad or lack formal, widely-accepted guidelines. In the dynamically changing fields of medicine, health, and science, up-to-date guidelines are often difficult to maintain.

### The Israeli context

A recent Israeli survey of a nationally representative sample of just over 7,000 7th–12th graders in 158 Ministry of Education schools found that among the 98.7% of adolescents who have Internet access, 52% had searched for health-related information online in the 12 months prior to the survey. While many information-seeking adolescents access OHI by going directly to specific health websites and portals, a much greater proportion (70%) seek information using search engines, primarily Google.co.il, Finder, and Kafe [41]. About one third (35%) of the adolescent Internet users reported being aware of the problematic nature and biases of OHI, but few view online information critically. Only 43% of OHI-seekers assess the accuracy of the information they find on the Internet. In a survey of Israeli primary care physicians, one-third (32.3%) expressed concerns about the quality of the information retrieved by their patients [42].

While the majority of adolescent Internet users in Israel seek OHI, there has been little, if any, assessment of the quality of Hebrew-language OHI, particularly on topics relevant to this population group [43]. The provision of high quality online information about oral contraceptive (OC) use is a priority for a number of reasons. Availability of quality information on OC has been shown to improve compliance with medical instructions, and avoid complications and misuse [44], and the Internet is an efficient and effective channel for delivery of sexual and reproductive health promotion messages [45]. In Israel about one-third of 12th grade girls have experienced coitarche [46]; over 40% of sexually active adolescents use OC [47], as much as 60% of adolescents harbor misconceptions about OC use [47], and about 15% of women under the age of 20 have sought termination of pregnancy [48]. Moreover, one-quarter of Israeli adolescents who searched online for health information looked for information about OC [41]. This study aims to 1) assess the accuracy/completeness, credibility, and usability of Hebrew online (non-user-generated) content on OC; 2) assess the correlation between these three parameters; and 3) examine the extent to which the quality of information and the correlation across the three parameters varies according to the type of information provider (e.g., HMO, promotional websites).

## Methods

### Search strategy

The selected websites were chosen based on the results of a national survey that identified web-sources commonly used by Israeli school adolescents when seeking OHI [41]. According to the survey, over 70% of students indicated having sought health information through Google.co.il, Finder, and Kafe. Five other search engines (Tapuz, Nana10, Walla, Bing, and Yahoo) were also mentioned in relation to searching for OHI, but less frequently. Therefore, several searches were conducted using identical search terms in Hebrew using Bing, Finder, Google, Kafe, Nana10, Walla, and Yahoo. Tapuz was excluded as it searches only within the Tapuz website itself.

We gathered the first 100 search results for each search term [various Hebrew permutations of birth control pill, birth control pills, pregnancy prevention pills, and contraceptives] on 6 search engines. The seventh search engine–Nana10, produced fewer than 30 results per search term and therefore all of Nana10’s results were added to the list. To increase the number of possible websites to be assessed and in order to avoid duplicates, the results were organized into four groups based on the type of search engine: 1) Google and Google-powered search engines–Finder and Kafe, 2) Yahoo-powered search engines–Yahoo and Walla, 3) Bing, and 4) Nana10. For each search term, we compared the results from the four search engine groups and excluded overlapping items. A list of 158 websites was compiled, after combining the results of the search of the six search-terms and excluding non-Hebrew websites. Of these, 27 were unique (i.e., not duplicates), accessible, active, and provided (non-user-generated) OC information on at least one webpage. This list contained the websites of two of the four major Israeli HMOs (Health Management Organizations – Kupat Holim), so the websites of the other two HMOs were added in order to assess the quality of all four HMOs. Thus, 29 websites were selected for evaluation. Within each website, text searches were conducted to identify all the pages included in the same domain name with at least one title and one paragraph with information on OC use. When available, non-OC related pages of the website (such as About pages, Contact pages, and pages with information on Privacy policy) were also evaluated to assess parameters of credibility (e.g., website’s funding, authors, and age policies) and usability (e.g., consistency in design and function).

### Website quality evaluation

The quality of each website was assessed on three key parameters: accuracy/completeness, credibility, and usability.

### Accuracy and completeness

Accuracy/completeness of the OC content was assessed based on 13 established content items selected by a family planning expert from contraception guidelines [49]. Items that were misperceived by Israeli physicians in a study on OC misconceptions [47] (perhaps due to inconclusive scientific evidence) were excluded, as we were unable to definitively classify the information as accurate or inaccurate. The final list included items such as efficacy (e.g., of 1000 women using the pill as prescribed, 3 will become pregnant within a year), effectiveness in the first year of use (e.g., about 8% of typical users initiating OC will become pregnant in the first year of use), safety, reversibility, suggested frequency of use (e.g., pills should be taken daily), STI protection (e.g., pills do not protect against STIs), side effects (e.g., headaches, nausea, vomiting, breast pain/tenderness, weight gain, fatigue, mood changes), dose (e.g., take one pill a day until the pack is finished), back-up contraceptive method (e.g., use a backup contraceptive until taking 7 consecutive pills, if missing a pill, if late starting a new pack, or in case of severe vomiting or diarrhea), or instructions in case of missing a pill (e.g., taking another pill within 12 hours will still prevent pregnancy). The 13 items were assessed on a 4-point scale [inaccurate information (−1), information not presented (0), information partially presented (1), and information complete and accurate (2)], and computed on a composite percentage scale ranging from 0 to 100%, with a score of 26 (the maximum possible for this 13-item scale) representing 100%.

### Credibility

The HONcode principles were developed to assess the credibility of OHI [35]. The eight HONcode principles address the web-author’s authoritativeness, the extent to which the information supports, rather than usurps, the doctor-patient relationship, stated privacy policy, attribution of the information to its sources, the extent to which the site backs up claims relating to benefits and performance, accessibility of the web-editor, financial disclosure, and advertising policy. Based on these original eight principles, a set of 14 questions was developed, each on a 3-point scale [parameter absent (1), parameter partially addressed (2), and parameter fully addressed (3)], and computed on a composite percentage scale ranging from 0 to 100%, with a score of 42 (maximum possible for the 14-item scale) representing 100%.

### Usability

Basic aspects of the websites’ usability were also assessed by applying an 11-item instrument selected from the Minervation Validation instrument (questions 2.1.2-2.1.5, 2.2.1-2.2.3, and 2.3.4) [50] and/or the University of Michigan “website evaluation checklist” (questions 12, 28, 30, 34, and 35) [51]. The items included aspects such as a “return to home page” option on all pages, consistency of functions and design, and appropriateness of content for the target adolescent audience (e.g., readability as assessed by amount and size of text, use of age-appropriate medical and scientific terminology). Each item was assessed on a 3-point scale [parameter absent (1), parameter partially addressed (2), and parameter fully addressed (3)], and computed on a composite percentage scale ranging from 0 to 100% with a score of 33 (maximum possible for the 11-item scale) representing 100%.

### Classification of websites

The websites were classified as belonging to one of the following 5 categories: a) HMOs (i.e., websites of the four leading Kupat Holim), b) health portals (i.e., websites that provide information on a wide scope of health and medical subjects), c) contraception-specific (i.e., websites dedicated primarily to informing the public on the subject of contraception), d) promotional and commercial (i.e., websites whose goal is to promote a certain product, service, or brand), e) life style and women’s websites (i.e., websites that provide information on contraception in the context of women’s wellbeing and child-rearing advice).

### Reliability analyses

All websites were evaluated by one of us (LF). Following analyses of the data and in response to a reviewer’s suggestion on a previous version of this report, six randomly selected websites (a total of 38 web pages) were independently assessed by YN and RS, and reassessed by LF. Some inter-rater variation was observed for accuracy/completeness and usability (Cronbach’s alpha coefficients–0.84 and 0.73, respectively; Additional file 1). A greater degree of variation was noted for the 15-item credibility scale, which revealed a Cronbach‘s alpha coefficient of 0.66. This value increased to 0.75 upon deletion of one item (“Is there a clear statement regarding the minimum age for the use or participation on the website?”). The credibility parameter for all 29 websites was therefore reanalyzed using the 14-item scale.

### Statistical analysis

Mean scores, standard deviations (SD), ranges, and percentages were calculated for all websites and for category-specific websites. Pearson correlation coefficients of the three parameters assessed were estimated for all, and for category-specific websites.

Statistical analyses were performed using SPSS 17.0.

## Results

### Accuracy/Completeness

Evaluation of individual scale items indicates that more than half (n=15–25, 51.7–86.2%) of the websites evaluated provided information regarding the efficacy, safety, reversibility, side effects, and general use (e.g., dose, when to start OC package), but many failed to provide complete information, or provided inaccurate instructions regarding what to do when a pill is missed and when to use back-up methods (Table 1).

**Table 1:** Frequency distribution of parameter items assessed for 29 Hebrew-language websites regarding information about OC

**Accuracy/Completeness**	**Inaccurate information (%)**	**Information not presented (%)**	**Information partially presented (%)**	**Information accurate and complete (%)**
OC efficacy	4 (13.8)	8 (27.6)	2 (6.9)	15 (51.7)
OC effectiveness	0	20 (69.0)	2 (6.9)	7 (24.1)
OC safety	0	12 (41.4)	0	17 (58.6)
Reversibility of OC	1 (3.4)	7 (24.1)	0	21 (72.4)
Indication to use OC daily	0	3 (10.3)	1 (3.4)	25 (86.2)
Indication that OC not protective against STIs	0	12 (41.4)	0	17 (58.6)
Side effects described	0	9 (31.0)	5 (17.2)	15 (51.7)
Instructs to take a pill daily until pack is finished; when to re-start use	0	6 (20.7)	2 (6.9)	21 (72.4)
When to take missed pill	1 (3.4)	12 (41.4)	2 (6.9)	14 (48.3)
Back-up method recommended during initial OC use	0	21(72.4)	2 (6.9)	6 (20.7)
Back-up method recommended if missing a pill	2 (6.9)	14 (48.3)	0	13 (44.8)
Back-up method recommended if late starting a new pack	21 (72.4)	0	2 (6.9)	6 (20.7)
Back-up method recommended in case of diarrhea or severe vomiting	0	18 (62.1)	2 (6.9)	9 (31.0)
**Credibility**	**Parameter absent (%)**	**Parameter partially addressed (%)**	**Parameter fully addressed (%)**	
Authorship disclosed	10 (34.5)	8 (27.6)	11 (37.9)	
Authorship expertise on the subject	12 (41.4)	3 (10.3)	14 (48.3)	
Authorship qualifications stated	10 (34.5)	2 (10.3)	17 (58.6)	
OHI reflects author’s opinion only	12 (41.4)	1 (3.4)	16 (55.2)	
OHI does not replace health professional advice	8 (27.6)	2 (6.9)	19 (65.5)	
Website purpose and intended audience stated	8 (27.6)	0	21 (72.4)	
Information about organization behind the website presented	11 (37.9)	0	18 (62.1)	
Privacy policy declared	13 (44.8)	1 (3.4)	15 (51.7)	
Date of creation and last modification	18 (62.1)	0	11 (37.9)	
Sources of health content cited	23 (79.3)	3 (10.3)	3 (10.3)	
OHI describes/refers to scientific evidence	18 (62.1)	4 (13.8)	7 (24.1)	
Several alternatives/treatments described	5 (17.2)	2 (6.9)	22 (75.9)	
Webmaster contact information available	1 (3.4)	0	28 (96.6)	
Sources of funding declared	17 (58.6)	0	12 (41.4)	
**Usability**	**Parameter absent (%)**	**Parameter partially addressed (%)**	**Parameter fully addressed (%)**	
Clear and legible layout of information	0	1 (3.4)	28 (96.6)	
Audience appropriate content	1 (3.4)	3 (10.3)	25 (86.2)	
Easy to identify links and buttons	0	0	29 (100)	
Links and buttons indicate where they lead	0	1 (3.4)	28 (96.6)	
Navigation clear and well structured	2 (6.9)	1 (3.4)	26 (89.7)	
Easy to find needed information	1 (3.4)	4 (13.8)	24 (82.8)	
Consistent function of navigational links	0	5 (17.2)	24 (82.8)	
Easy to return to homepage	0	0	29 (100)	
Easy to determine current location in the site	9 (31.0)	2 (6.9)	18 (62.1)	
Consistent site structure (categories or organization of pages)	1 (3.4)	4 (13.8)	24 (82.8)	
Consistent website design	4 (13.8)	0	25 (86.2)	

The mean score for accuracy/completeness was 50.9% (SD=30.1%) for all websites evaluated. Contraception-specific websites contained the most accurate and complete information (mean=66.7%) compared with a mean of 62.2% for health portals, 50.0% for HMOs and life style and women’s health websites, and 40.4% for promotional websites.

**Figure 1:**
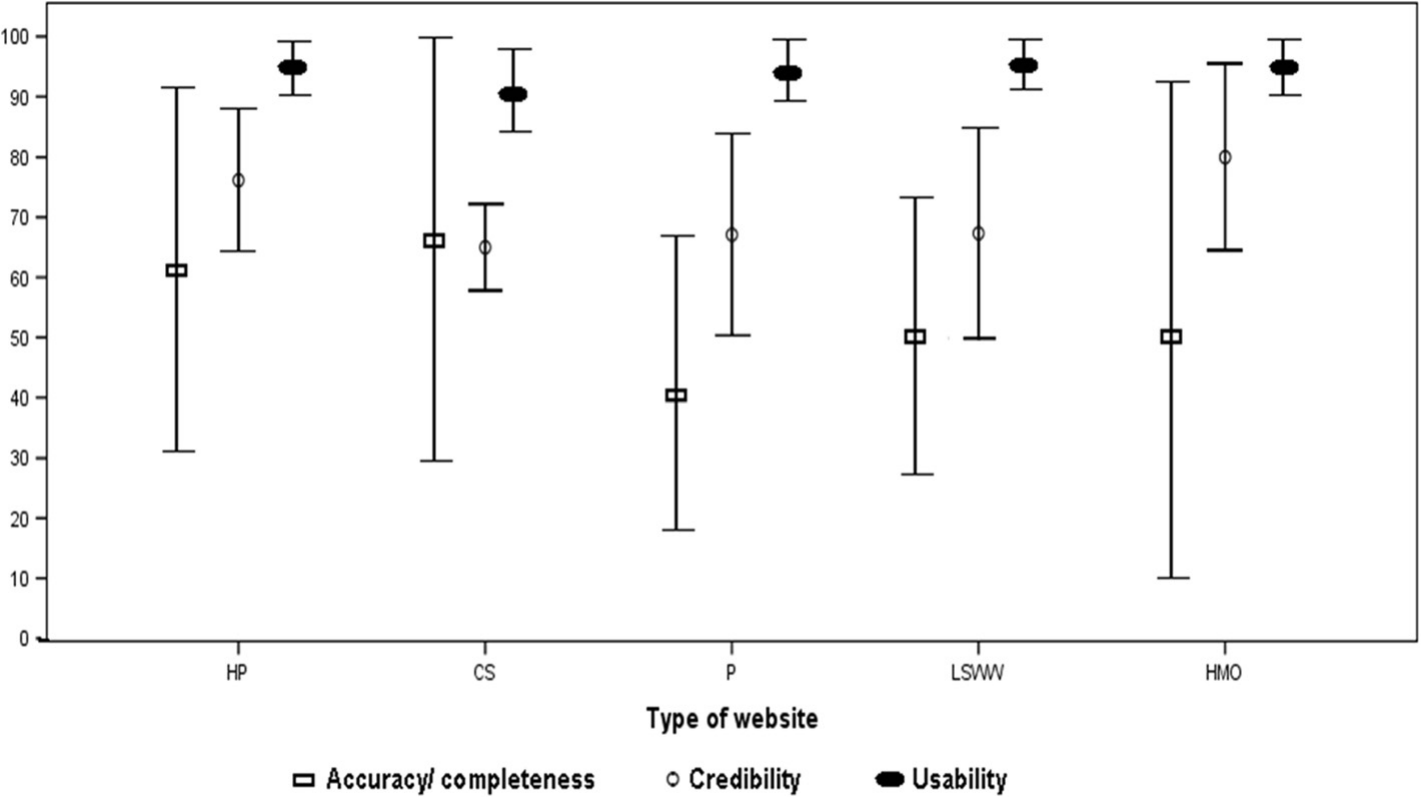
**Mean (SD) accuracy/completeness, credibility, and usability scores of Hebrew-language websites on oral contraceptives by website category.** CS=Contraception-Specific, HMO=Health Maintenance Organization, HP=Health Portals, LSWW= Life Style & Women’s Websites, P=Promotional.

As seen in Figure 1, variance of accuracy/completeness scores was highest for the HMO websites (SD=42.7%, range=8–92%), followed by promotional websites (SD=27.8%, range=12–89%), life style and women’s health websites (SD=24.9%, range=12–81%), health portals (SD=29.4%, range=31–100%), and contraception-specific websites (SD=37.8%, range=23–89%).

### Credibility

Two-thirds (n=18–19, 62.1–65.5%) of the websites stated that the health information they contained should not replace the advice of a health professional, and provided information about the organization that owns the website. Just over half (n=15–17, 51.7–58.6%) of the websites mentioned their content author’s qualifications and expertise, stated that information presented reflects only the opinion of the author, and presented their privacy policy. About three-quarters (n=21–22, 72.4–75.9%) of the websites contained information as to their intended audience and purpose, and provided information about different contraception methods. Nearly all websites had a contact e-mail address (n=28, 96.6%). Credibility parameters that were commonly missed included sources of funding, webmaster contact information, date of content creation and last modification, explicit reference to scientific evidence-based information, and references and citations (Table 1).

The mean score for credibility was 70.6% (SD=15.1%) for all websites evaluated. The HMOs’ websites contained the most credible information (80.0%), while contraception-specific websites had the lowest credibility score (65.0%). Health portals’ credibility (76.2%) was similar to that of the HMOs’ websites and higher than that of the promotional websites (67.1%) and life style and women’s health websites (67.3%).

For all websites, the lowest mean credibility score was 38% and the highest was 98%. Promotional websites (SD=16.8%, range=38–88%) and life style and women’s health websites (SD=17.5%, range=48–86%) exhibited the greatest variability followed by HMO websites (SD=15.5% range=62–98%). Health portals (SD=11.8%; range=60–88%) and contraception-specific websites (SD=7.2%, range=57–71%) showed the least variation in their credibility scores (Figure 1).

### Usability

Except for one item (location inside the website), usability items were commonly presented or addressed (n=18–29, 62.1–100%; Table 1). The average usability scores for all websites was 94.5% (SD=6.9%) and were virtually identical across the HMOs’ websites (96.3%), life style and women’s health websites (96.0%), health portals (95.5%), promotional websites (93.7%), and contraception-specific websites (90.0%).

As seen in Figure 1, the greatest variation in usability scores was noted among promotional websites (SD=8.4%, range=79–100%), followed by the HMOs’ websites (SD=7.5%, range=85–100%), contraception-specific websites (SD=7.5%, range=82–97%), health portals (SD=6.2%, range=85–100%), and life style and women’s health websites (SD=5.3%, range=88–100%).

### Inter-parameter correlations

Overall, low correlation coefficients were noted between credibility and accuracy/completeness (0.08), between credibility and usability (0.10), and between accuracy/completeness and usability (0.25); however, these varied across website categories. Among HMO websites, there was a strong positive correlation between credibility and accuracy/completeness (0.88), a weak positive correlation between usability and credibility (0.26), and an intermediate positive correlation between accuracy/completeness and usability (0.48). Among health portals, on the other hand, a strong negative correlation was noted between credibility and accuracy/completeness (−0.59), and weak correlations between credibility and usability (0.08), and accuracy/completeness and usability (0.16). Among the contraception-specific websites there was a weak negative correlation between credibility and accuracy/completeness (−0.24), a weak positive correlation between credibility and usability (0.17), and a strong positive correlation between accuracy/completeness and usability (0.92). Promotional websites revealed very weak correlations between credibility and accuracy/completeness (−0.08) and credibility and usability (−0.17), and accuracy/completeness and usability (0.05). Life style and women’s health websites showed little correlation between credibility and accuracy/completeness (0.08), a weak correlation between credibility and usability (0.29), a moderate positive correlation between accuracy/completeness and usability (0.50) (Table 2). None of the correlation coefficients reached statistical significance (*p* values ranged from 0.12 to 0.90).

**Table 2:** Correlation of quality parameters-accuracy/completeness, credibility, and usability, by website category

**Criteria**	**Website category**									
	**HMO N=4**		**Health portals**		**Contraception specific**		**Promotional**		**Life style & women’s health**	
	**HMO N=4**		**N=6**		**N=3**		**N=10**		**N=6**	
	**C**	**U**	**C**	**U**	**C**	**U**	**C**	**U**	**C**	**U**
Accuracy/completeness	0.88	0.48	−0.59	0.16	−0.24	0.92	−0.08	0.05	0.08	0.50
Credibility		0.26		0.08		0.17		A0.17		0.29

Note: *C* credibility, *U* usability.

## Discussion

The findings of this first study to evaluate the quality of online health information on OC use in Hebrew websites reveal wide variation in the quality of the websites evaluated. Overall, the accuracy/completeness and credibility scores of the evaluated websites were mid-range while usability scores were generally high. These dimensions also varied by website category, with contraceptive-specific websites exhibiting the highest accuracy/completeness scores and HMO websites showing the highest credibility and usability scores. Correlation coefficients between the three dimensions were generally low. None of the statistical comparisons of means or correlations reached significance, primarily due to the small sample size of websites in the various categories (e.g., the finite “sample” of four Kupat Holim websites).

Regardless of website category, all websites showed mid-scale accuracy/completeness scores, with the majority primarily failing to provide complete information. The commonly missing items included instructions on what to do if a pill is missed and when to use backup methods. Providing this information is relevant for OC users of all ages, but particularly for adolescent users, who frequently find it difficult to remember to take a pill every day. Poor dosage compliance constitutes one of the main reasons of method failure and teenage pregnancy among OC users [52].

As expected, subject-specific websites (i.e., contraceptive specific) yield better results in terms of accuracy/completeness, while promotional websites exhibited the lowest mean score. However, the mean score of accuracy/completeness was generally about 50% across websites of all categories. Notably, accuracy/completeness scores of the HMOs’ websites showed important variations. The low mean score for accuracy/completeness of the HMOs’ websites, coupled with a high standard deviation and a strong correlation (0.88) between credibility and accuracy/completeness scores, reflect a division between two sub-groups within the HMO website category. Two of the HMOs’ websites presented accurate, complete, and credible OC information, in sharp contrast with the information presented on the websites of the other two HMOs. While HMO websites with low OC quality information were added by the authors to the list of websites evaluated, HMO websites with the high OC quality information were explicitly mentioned by respondents in a national school survey [41] as online information sources, and they appeared consistently throughout the website selection procedures. This finding suggests that efforts to promote high quality online information by some HMOs seem to have been successful. Considering that about half to two-thirds of the traffic to higher scoring HMO websites arrives from Google, the impact of these two websites gains particular significance for Israeli adolescents seeking OC information.

Credibility parameters that were commonly absent included sources of funding, authorship disclosure, date of content creation and last modification, explicit reference to scientific evidence-based information, and references and citations. These parameters are anchors for contextualizing and comparing information; therefore, their appearance on the website is imperative. Knowing that the information retrieved is current is particularly important given the dynamic nature of medical practice and scientific knowledge. Furthermore, presenting the scientific and financial sources of information helps the reader identify the forces and interests influencing the content provided, and to act accordingly. In fact, websites that explicitly aim to promote health information, that is, health portals and the HMO websites, earned the highest credibility scores, in contrast to promotional websites and life style and women’s health websites, which earned lower scores. The low credibility of contraception-specific websites may prevent contraception information-seekers from fully benefitting from the highly accurate information they provide.

Usability scores were relatively homogeneous across websites. Standard guidelines and “pre-formatted” platforms facilitate designers’ compliance with existing usability scales. In contrast with the HMOs’ websites–the most usable websites, contraception-specific websites earned the lowest score for usability. This suggests that usability should not be relied upon as a sole indicator of the quality of information.

Overall, the correlation of the dimensions evaluated was very weak; however when assessed independently by website category, the HMO websites showed a strong correlation between accuracy/completeness and credibility (0.88). In contrast, health portals revealed a moderately strong negative correlation between accuracy/completeness and credibility (−0.59). The low correlation coefficients observed in the health portals category possibly reflects limited knowledge or expertise of health information providers in addressing the three quality dimensions evaluated, and suggest the need to advocate for regulating the design of online health content. At the same time, the discrepancy in the correlations emphasizes the need for providing adolescents with the skills necessary to assess the quality of the information retrieved, even from certified websites.

### HMO websites for youth

The four HMOs’ websites differ in their approach to adolescent health-information seekers. HMO-A’s website targets adolescents directly by dedicating a forum (one of nearly 40 forums) entirely to the adolescent audience. The forum’s specific information compensates to some extent for the insufficient and rather general scope of the editorial content for adolescents. Other than the forum, relevant information for youth, such as OC information, appears in the different website sections. No section, however, is designed specifically for adolescents or brings together information on health issues that are of greatest concern to young adults. At the time this review was conducted, HMO-A’s forums were only compatible with Internet Explorer and not with other popular browsers such as Mozilla, Firefox, and Google Chrome.

HMO-B’s website structure is guided by a concern for providing information about health topics (e.g., nutrition and physical activity) and situations requiring medical assistance (e.g., emergency medicine, pregnancy, and childbirth), rather than for directly addressing specific populations and audiences, such as adolescents. HMO-B’s website has a “Youth” section that features articles on topics such as smoking cessation, drug abuse, and medical screening checkups, although these articles seem to have in mind adults and parents more than adolescents.

HMO-C has a well-developed youth section on its website, featuring articles on a variety of topics, including drugs, mental health, nutrition, and teenage pregnancy. Yet, as with HMO-B, the articles seem to be written primarily for an adult audience, perhaps for concerned parents, and to a much lesser extent for teenagers seeking information on health issues. For example, in their youth section, there is a call to parents to provide adolescents with proper health information on sex, but no such information is actually provided.

HMO-D’s website approaches adolescent health information seekers by offering adolescents the option of asking a physician health questions through its website. Very few answers are published on the website, and interestingly, some of the questions were from parents, indicating that the online Q&A service is not exclusively for adolescents. Since the answers are not published, there is very little information on the website that addresses adolescents directly or even indirectly.

The effect of each particular approach on the process of health information seeking and health seeking behaviors requires further study. Interpretation and application of results of this website-evaluation study warrant a certain degree of caution. Although a comprehensive standard electronic evaluation form was designed and approved by the research team, the relatively low inter-rater reliability score for credibility suggests that credibility parameters might fluctuate from one page to another within a single website, or that the interpretation of these parameters is sensitive to the evaluators’ knowledge and beliefs. Second, the large number of websites and the great amount of online information on a seemingly infinite number of health-related subjects make it nearly impossible to generate a sample of websites that could be considered representative. To compensate, the evaluated websites were selected in such a way so as to resemble the array of OC websites that would be retrieved by an average Israeli adolescent health-information seeker. Finally, this evaluation was restricted to content posted by the website developers and excluded user-generated content (e.g., forums and social networks) that would require a different set of assessment tools [2].

## Conclusions

By implementing a systematic approach for the evaluation of online health information, our study identifies gaps between evidence-based information and the information available online regarding OC. Furthermore, the findings highlight, for the first time, the varied quality of Hebrew-language online information available about OC from different category websites, with generally low to medium scores in credibility and accuracy/completeness, and higher scores in usability. The weak correlation observed between the three quality dimensions assessed, particularly for promotional websites, suggests that quality guidelines should be established and promoted in order to improve the quality of OHI in general, and OC online information in particular. The findings also emphasize the need to strengthen the e-health literacy skills among online-information seekers. Adolescents should be provided with the skills needed to assess the quality of the online information available. Such skills would allow the information seeker to more effectively sort and understand the information retrieved, incorporate this information into their decision-making processes and consequently improve their health status. In addition, health care providers should be encouraged to support their patients in the process of selecting accurate and reliable health-related online resources.

## Abbreviations

OC: Oral contraceptives; OHI: Online Health Information.

## Competing interests

The authors declare that they have no competing interests.

## Authors’ contributions

YN and CL-Q designed the study. LF, CL-Q, and RS designed the instrument. LF collected the data and analyzed the websites. CL-Q performed the statistical analysis. LF, YN, and RS evaluated the websites. LF, CL-Q, and YN wrote the first drafts of the manuscript. All authors read and approved the final version of the manuscript.

## Authors’ information

Yehuda Neumark, PhD, MPH, is an Associate Professor of Epidemiology at the Braun School of Public Health & Community Medicine of Hebrew University-Hadassah in Jerusalem, Israel, where he teaches courses in epidemiology, research methods, and community-oriented health care, and directs the International Master of Public Health Program. His research focuses primarily on social, economic, and genetic variations in the epidemiology of alcohol and drug use and misuse, and the application of information and communication technologies for health promotion. He is the father of four and grandfather of six.

Lior Flum earned his master’s degree in Sociology and Anthropology at the Hebrew University of Jerusalem, Israel. Currently, he is a graduate student at the School of Education and Human Development at the University of Colorado – Denver, where he is completing a master’s degree in Information and Learning Technologies. His areas of interest include: e-Learning, social interactions and the web, e-Literacy, and online communication.

Catalina Lopez-Quintero earned her PhD in Public Health at the Braun School of Public Health & Community Medicine in the Hebrew University of Jerusalem, Israel, and is currently a post-doctoral fellow in the Department of Epidemiology and Biostatistics at Michigan State University. Her research focuses on identifying and understanding factors associated with drug use involvement and addiction, with the aim to translate her findings into health policy. Her other research interests include: e-health literacy, mental health, and HIV/AIDS among minority populations, children, and adolescents.

Ronny A. Shtarkshall holds a PhD in Physiology; a Diploma in Family Marriage and Sex Therapy (University of Pennsylvania); and is a registered social worker. He is a tenured faculty member at the Braun School of Public Health and Community Medicine of the Hebrew University and Hadassah, Jerusalem, Israel. His research interests include culture and sexuality, sexuality education, adolescents’ sexuality, immigrants’ health, multiple orgasms in women, and perceptions of sexual misconduct and violence.

## Supplementary Material

Additional file 1 **Inter-rater reliability estimates for analysis of accuracy/completeness, credibility, and usability parameters of Hebrew-language websites on oral contraceptives (Cronbach's Alpha if item deleted).** (DOC 53 kb) Click here for file
