# Supplementary material for: Quality of online health information about oral contraceptives from Hebrew-language websites
Source: Isr J Health Policy Res. 2012 Sep 24;1:38. doi: 10.1186/2045-4015-1-38 (PMC3475130; doi:10.1186/2045-4015-1-38)
Supplement: Additional file 1 — Inter-rater reliability estimates for analysis of accuracy/completeness, credibility, and usability parameters of Hebrew-language websites on oral contraceptives (Cronbach's Alpha if item deleted). (DOC 53 kb) [file 2045-4015-1-38-S1.doc]

Appendix 1: Inter-rater reliability estimates for analysis of accuracy/completeness, credibility, and usability parameters of Hebrew-language websites on oral contraceptives (Cronbach's Alpha if item deleted).

| **Accuracy/Completeness** | Alpha if item deleted |
| --- | --- |
| OC efficacy | 0.85 |
| OC effectiveness | 0.82 |
| OC safety | 0.83 |
| Reversibility of OC | 0.83 |
| Indication to use OC daily | 0.85 |
| Indication that OC not protective against STIs | 0.83 |
| Side effects described | 0.85 |
| Instructs to take a pill daily until pack is finished; when to re-start use | 0.84 |
| When to take missed pill | 0.82 |
| Back-up method recommended during initial OC use | 0.81 |
| Back-up method recommended if missing a pill | 0.82 |
| Back-up method recommended if late starting a new pack | 0.84 |
| Back-up method recommended in case of diarrhea or severe vomiting | 0.82 |
| **Credibility** |  |
| Authorship disclosed | 0.69 |
| Authorship expertise on the subject | 0.64 |
| Authorship qualifications stated | 0.66 |
| OHI reflects author's opinion only | 0.74 |
| OHI does not replace health  professional advice | 0.70 |
| Website purpose and intended audience stated | 0.73 |
| Information about organization behind the website presented | 0.68 |
| Privacy policy declared | 0.67 |
| Date of creation and last modification | 0.74 |
| Sources of health content cited | 0.71 |
| OHI describes/refers to scientific evidence | 0.70 |
| Several alternatives/treatments described | 0.77 |
| Webmaster contact information  available | 0.75 |
| Sources of funding declared | 0.71 |
| **Usability** |  |
| Clear and legible layout of information | 0.69 |
| Audience appropriate content | 0.66 |
| Easy to identify links and buttons | 0.68 |
| Links and buttons indicate where they take the user  lead | 0.70 |
| Navigation clear and well structured | 0.66 |
| Easy to find needed information | 0.69 |
| Consistent function of navigational links | 0.71 |
| Easy to return to homepage | 0.77 |
| Easy to determine current location in the site | 0.74 |
| Consistent site structure (categories or organization of pages) | 0.74 |
| Consistent website design | 0.75 |
